# Quality of coverage: Conformity measures for stereotactic radiosurgery

**DOI:** 10.1120/jacmp.v4i4.2506

**Published:** 2003-09-01

**Authors:** Q.‐R. Jackie Wu, B. W. Wessels, D. B. Einstein, R. J. Maciunas, E. Y. Kim, T. J. Kinsella

**Affiliations:** ^1^ Department of Radiation Oncology, Lerner Tower B181, 11100 Euclid Avenue, Case Western Reserve University School of Medicine and University Hospitals of Cleveland Cleveland Ohio 44106; ^2^ Department of Neurosurgery Case Western Reserve University School of Medicine and University Hospitals of Cleveland Cleveland Ohio 44106

**Keywords:** radiosurgery, dose volume histogram, conformity index

## Abstract

In radiosurgery, conformity indices are often used to compare competing plans, evaluate treatment techniques, and assess clinical complications. Several different indices have been reported to measure the conformity of the prescription isodose to the target volume. The PITV recommended in the Radiation Therapy Oncology Group (RTOG) radiosurgery guidelines, defined as the ratio of the prescription isodose volume (PI) over the target volume (TV), is probably the most frequently quoted. However, these currently used conformity indices depend on target size and shape complexity. The objectives of this study are to systematically investigate the influence of target size and shape complexity on existing conformity indices, and to propose a different conformity index–the conformity distance index (CDI). The CDI is defined as the average distance between the target and the prescription isodose line. This study examines five case groups with volumes of 0.3, 1.0, 3.0, 10.0, and 30.0 cm^3^. Each case group includes four simulated shapes: a sphere, a moderate ellipsoid, an extreme ellipsoid, and a concave “C” shape. Prescription dose coverages are generated for three simplified clinical scenarios, i.e., the PI completely covers the TV with 1 and 2 mm margins, and the PI over‐covers one half of the TV with a 1 mm margin and under‐covers the other half with a 1 mm margin. Existing conformity indices and the CDI are calculated for these five case groups as well as seven clinical cases. When these values are compared, the RTOG PITV conformity index and other similar conformity measures have much higher values than the CDI for smaller and more complex shapes. With the same quality of prescription dose coverage, the CDI yields a consistent conformity measure. For the seven clinical cases, we also find that the same PITV values can be associated with very different conformity qualities while the CDI predicts the conformity quality accurately. In summary, the proposed CDI provides more consistent and accurate conformity measurements for all target sizes and shapes studied, and therefore will be a more useful conformity index for irregularly shaped targets.

PACS number(s): 87.90.+y, 87.53.Ly

## INTRODUCTION

Radiosurgical targets are typically nonspherical, except for brain metastases. Some tumors exhibit more complex shapes than others. One goal of radiosurgery is to design a treatment plan in which the prescription isodose line covers the target with a minimal excess volume and a sharp dose fall‐off outside the target volume. However, planning or “mapping” the prescription isodose to a specific target shape can be a challenging task. Several different conformity indices have been reported to describe the conformity of the prescription isodose to the target volume. The PITV recommended in the Radiation Therapy Oncology Group (RTOG) radiosurgery guidelines is probably the most frequently quoted.^1^ The PITV is defined as the ratio of the prescription isodose volume (PI) to the target volume (TV). The RTOG guidelines define a ratio of 1.0–2.0 as per protocol and ratios in the range of 0.9–1.0 or 2.0–2.5 as minor variations. Knoos used a similar conformity index, RCI, when evaluating the conformity of radiation therapy plans.^2^ RCI is defined as the ratio of planning target volume to volume of prescription isodose line. Nedzi used a conformity index called treatment volume ratio (TVR) which was defined as the ratio of the target volume to the treatment volume.^3^ A modified PITV conformity index was suggested by Paddick, which takes into account the location of the prescription volume with respect to the target volume (see equation below).^4^

Although the PITV has been widely adopted as a benchmark for assessing radiosurgery conformity and outcomes, it, like other similar indices, depends on target size and shape. Accordingly, the PITV values for complex target shapes are often higher than those for simple target shapes. This effect has not yet been thoroughly studied in the literature. Kubo *et al*. has reported poor dose conformity from manual plans for complex target shapes using mMLC‐shaped static beam radiosurgery technique.^5^ He categorized the target shapes into three groups: “moderately irregular,” “irregular,” and “very irregular.” Using the same planning technique, the study found that moderately irregular and irregular targets had PITV ratios ranging from 1.5–2.0, while very irregular targets had PITV ratios ranging from 2.3–2.5. However, the study did not discuss the contribution of target complexity to the increased PITV values for highly irregular targets. Clinically, complex targets are harder to plan than more simply shaped targets. Hence, complex plans may inherently have slightly less conformity. But the contribution of target shape complexity to PITV values could be significant, making PITV values appear much worse than the actual quality of the plan.

The objective of this paper is to systematically investigate the contribution of target size and shape complexity to conformity index values. We also propose a distance‐based conformity measure, the conformity distance index (CDI), which is independent of target volume and shape. The CDI measures how closely the prescription isodose follows the target shape by returning the averaged distance between the two three‐dimensional (3D) surfaces.

## METHODS AND MATERIALS

### A. Target and coverage

**Figure 1:**
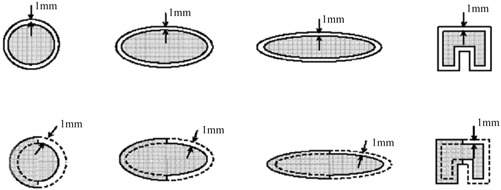
Target shapes and prescription isodose coverages. The upper row shows the prescription isodose lines for type 1 coverage, which is a 1 mm uniform expansion of the target volume. The lower row shows the prescription isodose lines (dashed line) for type 3 coverage, which under‐covers the left half of each target by 1 mm and over‐covers the right half of each target by 1 mm.

In order to study the conformity index systematically, we create simulated targets to represent the broad range of treatment volumes, sizes, and shapes seen in clinical practice. The simulated targets have well‐defined borders, eliminating the *in vivo* ambiguity introduced by target segmentation from computed tomography (CT) or magnetic resonance (MR) images. A total of five volumes, 0.3, 1.0, 3.0, 10.0, and 30.0 cm^3^, are studied. For each of these volumes, four different shapes are simulated. As shown in Fig. 1, these shapes include a sphere, a moderate ellipsoid, an extreme ellipsoid, and a concave “C” shape. These shapes resemble various clinical lesions, and similar shapes have been used to evaluate and compare the dosimetry of radiosurgery techniques by several other authors.^6^ ^–^ ^9^ The moderate ellipsoid has an elongation ratio of 1:1:1.6 for its three major axes. The extreme ellipsoid has an elongation ratio of 1:1:5, which simulates a “finger”‐like target. These size and shape combinations result in a total of 20 targets, as shown in Table I.

In clinical practice, a limited amount of over‐coverage of surrounding normal tissues and/or under‐coverage of the target is considered acceptable so that a treatment may be delivered with a reasonable number of isocenters or segments (intensity modulated plans).^8^ ^,^ ^10^ ^,^ ^11^ Overall, we assume that the prescription isodose can be 1 or 2 mm off from the TV for clinical cases. In this study, we simulate three simplified types of coverage:

(1) mm uniform over‐coverage: PI is the 3D uniform expansion of the TV by 1 mm or PI completely covers the TV with a 1 mm margin;
(2) mm uniform over‐coverage: PI is the 3D uniform expansion of the TV by 2 mm;
(3) Mixed under‐coverage and over‐coverage: PI over‐covers half of the TV by 1 mm and under‐covers half of the TV by 1 mm.

More specifically, condition 3 was generated in the following way: First, starting with coverage type 1, we split the TV and PI into two equal halves along one of the symmetrical axes. Second, for one of the halves, we simply reassigned the PI as TV and vice versa. Hence, we effectively generated a mixed‐coverage simulation with 1 mm over‐coverage to the normal tissue from the original half and 1 mm under‐coverage of the target from the other half. It is true that this changed the target volume slightly. However, the volumes we use in this study represent a volume group rather than a particular size. Therefore, small changes are not as critical as they would otherwise be. Figure 1 also shows the simulated target and prescription isodose coverage. This dose coverage was generated analytically to ensure that the prescription isodose coverage conditions were of identical quality for all targets, allowing an unbiased comparison on conformity parameters. By using analytical coverage and simulated targets, we also eliminated any uncertainty of the dose distribution caused by planning variations over the broad range of target volumes.

**Table I:** Conformity parameters of simulated targets.

		Dose coverage type 1		Dose coverage type 2		Dose coverage type 3		
Target type	Target volume (cc)	PITV	CDI (mm)	PITV	CDI (mm)	PITV	${\text{CI}}_{\text{PADDICK}}$	CDI (mm)
sphere	0.3	1.91	0.99	3.25	1.95	1.00	0.47	0.90
	1.0	1.57	1.00	2.31	1.98	1.00	0.61	0.93
	3.0	1.38	1.00	1.83	1.99	1.00	0.71	0.95
	10.0	1.24	1.00	1.52	1.99	1.00	0.80	0.96
	30.0	1.16	1.00	1.34	2.00	1.00	0.85	0.98
ellipsoid I	0.3	1.93	0.98	3.31	1.93	1.00	0.46	0.90
	1.0	1.58	0.98	2.35	1.95	1.00	0.60	0.93
	3.0	1.38	0.99	1.85	1.96	1.00	0.70	0.95
	10.0	1.25	0.99	1.53	1.97	1.00	0.79	0.96
	30.0	1.17	0.99	1.35	1.97	1.00	0.85	0.97
ellipsoid II	0.3	2.14	0.92	3.84	1.91	1.00	0.40	0.88
	1.0	1.72	0.92	2.68	1.85	1.00	0.54	0.90
	3.0	1.47	0.92	2.05	1.87	1.00	0.65	0.90
	10.0	1.30	0.92	1.65	1.84	1.00	0.75	0.91
	30.0	1.21	0.92	1.44	1.84	1.00	0.82	0.91
“c” shape	0.3	2.64	1.04	5.11	2.03	1.00	0.30	0.98
	1.0	1.91	1.03	3.13	2.03	1.00	0.47	0.98
	3.0	1.56	1.02	2.26	2.02	1.00	0.61	0.99
	10.0	1.34	1.02	1.75	2.02	1.00	0.73	0.99
	30.0	1.23	1.01	1.48	2.02	1.00	0.81	0.99

### B. Conformity evaluation

Three conformity parameters are calculated for each of the target‐coverage combinations. The RTOG conformity index PITV is defined as

(1) $$\text{PITV}=\frac{\text{PI}}{\text{TV}},$$

where PI is the volume of the prescription isodose line, and TV is the target volume. The conformity index, ${\text{CI}}_{\text{Paddick}}$, suggested by Paddick, is another commonly utilized radiosurgery conformity measure.^4^ It is defined as

(2) $${\text{CI}}_{\text{Paddick}}=\frac{{\text{TV}}_{\text{PI}}}{\text{PI}}X\frac{{\text{TV}}_{\text{PI}}}{\text{TV}}=\frac{{\text{TV}}_{\text{PI}}^{2}}{\text{PI}\times \text{TV}},$$

where ${\text{TV}}_{\text{PI}}$ is the target volume within the prescribed isodose volume PI. In this formula, ${\text{CI}}_{\text{Paddick}}$ becomes the inverse of PITV when the prescription isodose fully covers the target. A perfect plan would have ${\text{TV}}_{\text{PI}}=\text{TV}=\text{PI}$ and yield a ${\text{CI}}_{\text{Paddick}}$ of 1.0 as well as a PITV of 1.0. Other conformity formulas are very similar to PITV or ${\text{CI}}_{\text{Paddick}}$, hence they will not be discussed further in this paper.

In this study, we introduce a different type of conformity parameter denoted as the conformity distance index (CDI). The CDI measures the average distance between the prescription isodose and the target contour. Since calculating the norm and searching the distance from one point to a curved surface in 3D space is complex and time consuming, an approximation using the volume and surface of the TV and PI is implemented in this study. This approximation will be very close to the true CDI since the isodose and target surfaces from the radiosurgery techniques are continuous and smooth. Mathematically, the CDI is defined as

(3) $$\text{CDI}=\frac{{\text{NT}}_{\text{PI}}+(\text{TV}-{\text{TV}}_{\text{PI}})}{\frac{1}{2}\left(S_{\text{PI}}+S_{\text{TV}}\right)}=\frac{\left(\text{PI}-{\text{TV}}_{\text{PI}}\right)+\left(\text{TV}-{\text{TV}}_{\text{PI}}\right)}{\frac{1}{2}\left(S_{\text{PI}}+S_{\text{TV}}\right)},$$

where $S_{\text{PI}}$ and $S_{\text{TV}}$ are surfaces of the PI and TV, ${\text{TV}}_{\text{PI}}$ is the target volume receiving the prescription isodose or higher, and ${\text{NT}}_{\text{PI}}$ is the normal tissue volume receiving the prescription dose or higher. ${\text{NT}}_{\text{PI}}$ is the geometrical subtraction of the PI from TV, which evaluates the over‐coverage to the normal tissue. The second term of the above equation, $\text{TV}–{\text{TV}}_{\text{PI}}$, is also a geometric subtraction and evaluates the under‐coverage of the target volume.

### C. Comparison with clinical cases

We randomly selected seven clinical cases from our radiosurgery database to compare their conformity parameters. All these cases were planned using Gamma Knife radiosurgery technique. In all cases, more than 98% of the TV received the prescription dose or higher, making the ${\text{CI}}_{\text{Paddick}}$ very close to the PITV Therefore, only the PITV and the CDI were calculated. Among these cases, the volumes ranged from 0.14 cm^3^ to 8.6 cm^3^. One case was an acoustic schwannoma, which exhibits sharp corners in shape. The other cases included meningioma and metastases, which are closer to ellipsoid shapes. The PITVs were calculated directly from the Dose‐Volume‐Histogram (DVH). The CDI, however, could not be calculated directly from the GammaPlan™ as it does not provide surface area information. We approximated the target and prescription isodose volumes as ellipsoids to calculate the surfaces. For each target, the major axes were measured to get the elongation ratio and then fitted into the formula to make an ellipsoid that matched the volume. The measurements are listed in Table II.

**Table II:** Conformity parameters of clinical cases.

Case	Diameter a/b/c (mm)			Volume (cc)	PITV	CDI (mm)
1	18.0	18.0	22.5	3.82	1.15	0.45
2	5.1	5.1	10.5	0.14	1.51	0.44
3	14.3	14.3	17.0	1.82	1.30	0.70
4	3.1	3.1	3.5	0.02	5.03	1.10
5	23.5	23.5	29.7	8.59	1.47	1.71
6	19.3	19.3	24.2	4.72	1.32	1.00
7	12.8	12.8	20.0	1.72	1.52	1.09

## RESULTS

**Figure 2:**
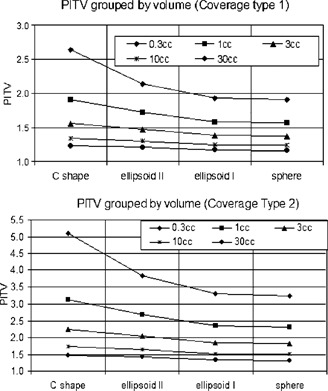
PITV grouped by volumes for coverage type 1 (top) and for coverage type 2 (bottom).

**Figure 3:**
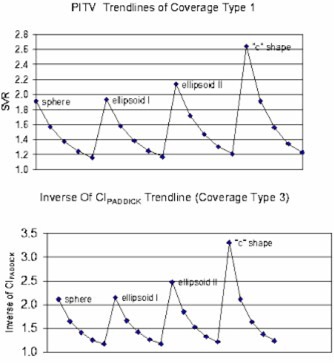
(Color) (top). PITV for coverage type 1. (bottom). Inverse of ${\text{CI}}_{\text{Paddick}}$ trendlines for coverage type 3.

Table I shows the conformity parameters for all of the simulated target‐coverage combinations. Since ${\text{TV}}_{\text{PI}}=\text{TV}$ for coverage types 1 and 2, the ${\text{CI}}_{\text{Paddick}}$ is the same as the PITV and its value is the inverse of the PITV. Therefore, the ${\text{CI}}_{\text{Paddick}}$ data are not listed in the table. For coverage type 1, where PI is the 1 mm uniform expansion of TV, the CDI value ranges from 0.92 to 1.04 mm. For coverage type 2, the CDI value ranges from 1.82 to 2.03 mm. The PITV values, however, fluctuate as target size and shape change. To analyze the fluctuation, we broke the PITVs into five volume groups as shown in Fig. 2. The smallest volume group (0.3 cm^3^) is the highest curve and the largest volume group (30 cm^3^) is the lowest curve. Within the same volume group, the PITV has the lowest value for the sphere and the highest value for the “C” shape, indicating a trend toward higher index values for more complex shapes. The PITV curve for the moderate ellipsoid shape is very close to that of the sphere. Figure 3(a) shows the PITV values grouped by corresponding shape. There are four “waves,” each indicating a shape group. Within each “wave,” the smallest volume has a peak index value and the largest volume has a valley index value. Additionally, the PITV curve has a global trend of moving upward as shape complexity increases.

For coverage type 3, the PITV values are a constant of 1.0 for all cases since the amount of over‐coverage of normal tissue and under‐coverage of target cancel out. The ${\text{CI}}_{\text{Paddick}}$ ranges from 0.303 to 0.854, and the CDI ranges from 0.88 cm to 0.99 mm. Figure 3(b) shows the inverse of the ${\text{CI}}_{\text{Paddick}}$ of all the targets for this type of coverage. The trend is very similar to the PITV trendline in Fig. 3(a).

Table II lists the data from the seven clinical cases. Excluding case #4, the PITV ranges from 1.15 to 1.52, while the CDI ranges from 0.45 to 1.71 mm. There is no correlation between the PITV values and CDI values. For example, cases #2 and #5 have similar PITVs (1.51 vs. 1.47). However, case #2 has a much smaller CDI (0.44 mm) than case #5 (1.71 mm). Cases #1 and #2 have similar CDI values (0.45 mm) while their PITVs are 1.15 vs. 1.51. Case #4 has a very small volume of 0.02 cm^3^ and a significantly higher PITV value of 5.03 with a CDI of 1.1 mm.

## DISCUSSION

**Figure 4:**
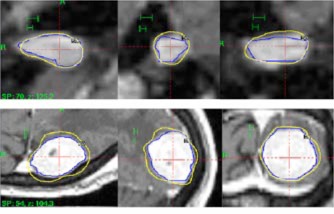
Prescription isodose distribution in axial, coronal, and sagittal views. The upper row shows the prescription isodose for case #2 and the lower row for case #5.

In this study, we found that both volume and shape complexity can have significant effects on conformity values. As shown in Figs. 2 and 3, the PITV and ${\text{CI}}_{\text{Paddick}}$ tend to have higher values for smaller or more complex targets even when the prescription dose coverage is identical to other targets with larger volumes or simpler shapes. These findings are consistent with the data from clinical studies summaries in Table II. For example, cases #2 and #5 have similar PITVs (1.51 vs. 1.47). However, case #2 has a much smaller CDI (0.44 mm) than case #5 (1.71 mm). Figures 4(a) and 4(b) show the prescription isodose coverage of case #2 and #5, respectively. Each picture also has 2 mm and 1 mm scale bars to indicate the magnification of each picture. Clearly, case #2 is a more conformal plan than case #5. In the mean time, case #2 has a smaller volume and larger elongation ratio. Therefore target complexity influences the PITV value significantly and makes conformity quality appear much worse. However, the CDI accurately predicts how closely the PI follows the target contour. In summary, the current conformity indices such as PITV would provide misleading results of the conformity quality of a treatment plan when examining small target sizes or complex target shapes. The CDI, on the other hand, accurately predicts how closely the PI is following the target contour for any target shape and size.

The conformity distance index, CDI, proposed in this study directly measures the distance deviation of PI from TV The CDI value of 1.0 mm indicates that the average distance from the prescription isodose line to the target surface is about 1 mm. We believe that this provides a more straightforward and useful interpretation of conformity than other common indices. However, the comprehensive evaluation of a treatment plan should be based not only on the conformity measures but also on the dose volume histogram (DVH) of the target, normal tissues, existing critical structures, and the dose distribution overlaid on anatomical images.

## CONCLUSION

This study has demonstrated that currently used conformity measures are dependent on the target size and shape. To overcome this limitation, we proposed a distance‐based conformity index (CDI) in which the conformity of PI to TV is measured as the average distance between the two surfaces. Our analysis indicated that CDI is independent of target size and shape. Therefore it can be used to achieve more accurate measurements as a conformity index for small and irregular targets than those of currently used conformity indices.

## ACKNOWLEDGEMENTS

This work is supported in part by a grant from the Whitaker Foundation (R‐00‐0427). The authors would like to thank Bonnie Hami for her help in editing the manuscript.
